# Association of sodium-glucose cotransporter 2 inhibitors with cardiovascular outcome and safety events: A meta-analysis of randomized controlled clinical trials

**DOI:** 10.3389/fcvm.2022.926979

**Published:** 2022-10-14

**Authors:** Chen Gong, Shi-Chun Shen, Ke Zhang, Lei Zhou, Jun-Jie Shen, Jia-Ying Zhao, Sheng-Gang Ding, Li-kun Ma, Hui Gao

**Affiliations:** ^1^Department of Pediatrics, The First Affiliated Hospital of Anhui Medical University, Hefei, China; ^2^Department of Cardiology, The First Affiliated Hospital of USTC, Division of Life Sciences and Medicine, University of Science and Technology of China, Hefei, China; ^3^Department of Laboratory Medicine, The First Affiliated Hospital of Anhui Medical University, Hefei, China; ^4^Department of Cardiology, Jinshan Hospital of Fudan University, Shanghai, China; ^5^Second School of Clinical Medicine of Anhui Medical University, Hefei, China

**Keywords:** sodium-glucose cotransoporter-2 inhibitors, cardiovascular risks, diabetes, atherosclerotic cardiovascular disease, heart failure

## Abstract

**Background:**

The clinical benefit of sodium-glucose cotransporter 2 (SGLT2) inhibitors for preventing and treating cardiovascular events remains controversial. We aimed to study the effect of SGLT2 inhibitors on cardiovascular outcomes and safety events, giving particular attention to the benefits in subgroups of patients with different diseases.

**Method:**

Randomized controlled trials (RCTs) reporting cardiovascular outcomes following the administration of SGLT2 inhibitors and placebo were included in this study. Cardiovascular outcomes included all-cause death, major adverse cardiovascular events (MACEs), cardiovascular (CV) death, myocardial infarction (MI), stroke, and hospitalization for heart failure (HHF). We also focused on the cardiovascular benefits of SGLT2 inhibitor application in subgroups of patients with different diseases, including type 2 diabetes (T2D), heart failure (HF), high risk of atherosclerotic cardiovascular disease (ACD), diagnosed ACD, and chronic kidney disease (CKD). Safety events associated with SGLT2 inhibitors, including acute kidney injury (AKI), diabetic ketoacidosis (DKA), hypoglycemia, urinary tract infection, thromboembolic event, bone fracture, volume depletion, and amputation, were also reported.

**Results:**

This meta-analysis included 15 RCTs with 78,212 participants. SGLT2 inhibitors reduced the risk of all-cause death (RR 0.89; 95% CI: 0.85–0.94; I2 = 32%; *p* < 0.01), CV death (RR 0.87; 95% CI: 0.82–0.93; I2 = 11%; *p* < 0.01), MACEs (RR 0.89; 95% CI: 0.84–0.94; I2 = 46%; *p* < 0.01), HHF (RR 0.70; 95% CI: 0.66–0.74; I2 = 0%; *p* < 0.01), and AKI (RR 0.81; 95% CI: 0.73–0.90; I2 = 0%; *p* < 0.01) but increased the risk of DKA (RR 2.56; 95% CI: 1.72–3.80; I2 = 0%; *p* < 0.01). However, no apparent benefit in MI and stroke was observed between the SGLT2 inhibitor and control groups. SGLT2 inhibitors reduced the risk of all-cause death, MACEs, CV death, and HHF in diabetic patients; reduced the risk of all-cause death, MACEs, CV death, MI, and HHF in primary prevention; reduced the risk of all-cause death, CV death, and HHF in patients with ACD and HF; and reduced the risk of MACEs, CV death, and HHF in patients with CKD.

**Conclusion:**

SGLT2 inhibitors have a positive effect in reducing the risk of all-cause death, CV death, MACE, HHF, and AKI and increasing the risk of DKA. The application of SGLT2 inhibitors in the primary prevention of ACD also has certain clinical benefits in reducing MI.

**Systematic review registration:**

[https://www.crd.york.ac.uk/prospero/], identifier [CRD42022306490].

## Introduction

Sodium-glucose cotransporters (SGLTs) is a family of membrane proteins with a similar core structure involved in the transport of glucose, amino acids, and some ions *in vivo* (1). SGLTs includes a family of glucose transporters found in the intestinal mucosa of the small intestine (enterocytes) and the proximal tubules of the nephron [proximal convoluted tubule (PCT) and proximal straight tubule (PST)]. SGLT1 was first cloned in 1987; it has a low ability to transport glucose and is mainly found in the intestine, trachea, heart, and kidney (2, 3). SGLT2 is mainly distributed in the PCT epithelium of the kidney. Although SGLT2 has a low affinity for glucose, it has a high ability to transport glucose. In the kidney, 98% of the glucose filtered by the glomerulus is reabsorbed by SGLT2 in the PCT (4). SGLT2 inhibition reduces glucose absorption in the proximal tubule to reduce blood glucose levels. Therefore, SGLT2 inhibitors are approved as a new type of hypoglycemic drug for treating type 2 diabetes (5).

As diabetes is closely related to cardiovascular diseases, the U.S. Food and Drug Administration (FDA) requires that hypoglycemic treatments be safe for the cardiovascular system (6). Several large randomized controlled trials (RCTs) are aimed at revealing the association between SGLT2 inhibitors and cardiovascular risks. DECLARE-TIMI58, an RCT with the largest sample size to date, was designed to investigate the association between dapagliflozin and cardiovascular risks in participants with atherosclerotic cardiovascular disease (ACD) or at high risk for ACD (7). Compared with the control group, dapagliflozin significantly reduced the risk of cardiovascular (CV) death by 17% and reduced the risk of hospitalization for heart failure (HHF) by 27%. However, dapagliflozin did not reduce the risk of major adverse cardiac events (MACEs) or myocardial infarction (MI). EMPA-REG OUTCOME, a multicenter, double-blind, randomized controlled clinical trial, was designed to investigate the effect of empagliflozin on cardiovascular morbidity and mortality in patients with type 2 diabetes (T2D) or high risks for cardiovascular events (8). The results from the EMPA-REG OUTCOME showed that empagliflozin reduced the risks of MACE, CV death, HHF, and all-cause death. In recent years, studies have noted that SGLT2 inhibitors exhibit a good hypoglycemic effect, reduce the risks of ACD, and have potential benefits in heart failure (HF) (9). Zelniker et al. performed a meta-analysis that included three large clinical RCTs to analyze cardiovascular outcomes in patients with T2D who were administered SGLT2 inhibitors (9). They found that SGLT2 inhibitors had a moderate benefit on patients with T2D and ACD. Regardless of whether these subjects had ACD or HF, SGLT2 inhibitors reduced the risk of HHF and kidney disease. A recently published meta-analysis including 75,000 subjects revealed that use an SGLT2 inhibitor as a hypoglycemic therapy reduces the rate of HHF, of which empagliflozin has the best effect in reducing the risk of all-cause death and CV death (10). Although 64 RCTs were included in their study, some trials only reported safety events and changes in laboratory indicators in patients with T2D, which did not comprehensively reflect the risk of cardiovascular outcomes. In an assessment including two large clinical RCTs, EMPEROR-REDUCE and DAPA-HF, Zannad et al. noted that SGLT2 inhibitors can prevent the recurrence of HHF in patients with HF with reduced ejection fraction (EF) (11). EMPEROR-PRESERVED, a randomized, double-blind, controlled clinical trial, included 5,988 HF patients with an EF greater than 40% and NYHA class 2–4 (12). In this trial, SGLT2 inhibitors were shown to reduce HHF effectively, and this finding was observed in both diabetic and non-diabetic patients. However, a recent RCT, PRESERVED-HF, aimed to explore the clinical benefits of SGLT2 inhibitors in patients with heart failure with reduced ejection fraction (HFrEF) and found that the HFF rate was 5.6% in both the SGLT2 inhibitor-treated and control groups (13). Compared with EMPEROR-PRESERVED, the sample size of PRESERVED-HF is smaller. The different types of SGLT2 inhibitors used in the two trials may cause conflicting results. The results of the RCTs and meta-analyses mentioned above appear to be inconsistent, which prompted us to investigate the cardiovascular benefits of SGLT2 inhibitors further and explore whether these therapies can also be used for the treatment of HF. This study focuses on the cardiovascular risk of SGLT2 inhibitors for the overall enrolled population. We also focused on the cardiovascular impact of SGLT2 inhibitors in specific subgroups, including patients with T2D, patients with a high risk of ACD, and those diagnosed with ACD, HF, and CKD.

Although SGLT2 inhibitors appear to have the potential to reduce cardiovascular risk, it is undeniable that they also have certain safety concerns. Because SGLT2 inhibitors prevent renal tubular reabsorption of glucose, high glucose levels in the urine can lead to urinary tract infections and diabetic ketoacidosis (DKA). In 2013, SGLT2 inhibitors were approved as a class of oral drugs for the treatment of T2DM in the UK. However, two years later, SGLT2 inhibitors were observed to cause severe or even fatal DKA. Among them, some cases are associated with the misadministration of SGLT2 inhibitors in T1DM patients. The causes of DKA induced by SGLT2 inhibitors in T2DM may be related to higher glucagon levels, decreased inhibition of lipolysis and ketogenesis caused by reduced daily insulin requirements, and decreased urinary ketoexcretion (14, 15). Moreover, there are also reports of adverse events, such as bone fractures and amputations, after using SGLT2 inhibitors (7, 8). Therefore, in this study, we also analyzed the safety events after the use of SGLT2 inhibitors to clarify the contraindications for SGLT2 inhibitor administration to avoid clinical risks.

## Materials and methods

This meta-analysis was performed based on the Preferred Reporting Items for Systematic Reviews and Meta-Analyses (PRISMA) checklist and followed PRISMA guidelines (16). The registration number of the protocol of this meta-analysis for the PROSPERO database^1^ was CRD42022306490.

### Search strategy and inclusion criteria

This meta-analysis was designed according to the participants, intervention, comparison, outcome, study design (PICOS) principle. This meta-analysis was based on the following parameters: P = adults (above 18 years old); I = SGLT2 inhibitor; C = placebo, O = all-cause death, major adverse cardiovascular events (MACEs), cardiovascular (CV) death, myocardial infarction (MI), stroke, and HHF; S = RCT.

Two authors independently searched PubMed, Google Scholar, ClinicalTrials.gov, and the Cochrane Library from the inception of each of the above databases up to January 25, 2021 without language restrictions. The combination of the following search terms was used to search eligible RCTs in the above databases: SGLT2 inhibitor, canagliflozin, dapagliflozin, empagliflozin, ertugliflozin, sotagliflozin, heart failure (HF), ACD, coronary heart disease (CHD), myocardial infarction (MI), cardiovascular disease, and RCT.

### Data extraction and assessment of methodological quality

Two authors independently extracted the characteristics and assessed the methodological quality of each RCT. Disagreement were resolved by discussion until consensus was reached or by consulting a third author. In each included RCT, the trial name, first author, year of publication, recruitment period, sample size, interventions, duration of follow-up, trial registration number, the character of participants in every study, trial location, and study design were recorded.

The assessment of methodological quality was conducted following the Cochrane Collaboration risk of bias tool as follows: random sequence generation, allocation concealment, participant and personnel blinding, blinding of outcome assessment, incomplete outcome data, no selective outcome reporting, and other sources of bias (17). The risk of bias of each RCT was rated as low, high, or unclear.

### Cardiovascular outcomes and safety events

The cardiovascular outcomes included all-cause death, MACEs, CV death, MI, stroke, and HHF. MACE was defined as a composite of CV death, non-fatal MI, and non-fatal stroke. If the RCT did not report the occurrence of non-fatal MI and non-fatal stroke but reported the number of MI and stroke, then the latter events were extracted in this meta-analysis.

Safety events included acute kidney injury (AKI), DKA, hypoglycemia, urinary tract infection, thromboembolic event, bone fracture, volume depletion, and amputation.

### Subgroup analysis

To further analyze the cardiovascular risks of SGLT2 inhibitors in specific populations, we divided the included participants into subgroups as follows: patients with T2D, HF, high risk of ACD, diagnosed ACD, and CKD. If one RCT enrolled subjects with diagnosed CAD or high risk of CAD, the RCT was included in the corresponding subgroup according to the largest number of people at a certain development stage of CAD.

### Statistical analysis

We calculated RR values and 95% confidence intervals for prespecified cardiovascular outcomes and safety events. *I*^2^ and *P*-values were used to detect heterogeneity of inclusion in RCTs. An *I*^2^ value less than 50% or a *p*-value for heterogeneity greater than 0.1 was considered to indicate low heterogeneity, and an *I*^2^ value greater than 50% or a *p*-value for heterogeneity less than 0.1 was considered to indicate high heterogeneity. A fixed-effects model was used, and sensitivity analyses were performed to explore the source of heterogeneity when heterogeneity was high. Otherwise, a random-effects model was used. The statistical tests calculated by the fixed-effect model and random-effect model were compared to verify the robustness of our results. We detected publication bias using funnel plots and explored sources of publication bias using clip-and-fill. A *P*-value for an effect less than 0.05 was considered significant. RStudio (Version 1.4.1717) was used to calculate the statistical tests and generate funnel plots and forest plots to present the data.

## Results

**FIGURE 1:**
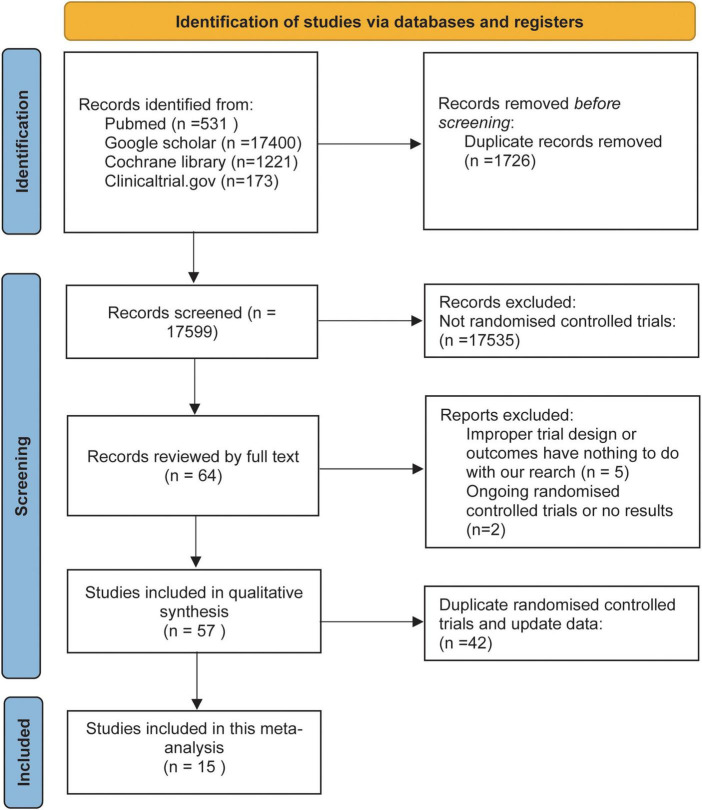
Flow diagram of the study selection process.

The initial literature search retrieved 19325 articles. A total of 19261 articles were excluded based on the title and abstract. Fourteen RCTs were finally deemed eligible and included in this meta-analysis after full-text review. Figure 1 shows our process for conducting a literature search and finalizing the included literature.

The study included 78,212 subjects, of whom 42,385 received SGLT2 inhibitors and 35,827 received placebo. These subjects included those with T2D, HF, high risk of ACD, diagnosed ACD, and CKD.

Table 1 shows the characteristics of the 14 included RCTs.

**TABLE 1:** Main characteristics of included RCTs.

Author-year	Trial registration	Location	Participants	Type of SGLT2 inhibitors/ control	No. SGLT2 inhibitors/ control	Follow-up duration, weeks	Endpoints assessed	Study design
Zinman et al. (8)	NCT01131676	worldwide	T2D	Empagliflozin/placebo	4687/2333	161.6	All-cause death, MACE, CV death, HHF, MI, stroke	Multicenter, double-blind RCT
Neal1 et al. (18)	NCT01032629	worldwide	T2D	Canagliflozin/placebo	2888/1442	295.9	All-cause death, MACE, CV death, HHF, MI, stroke	Multicenter, double-blind RCT
Neal et al. (18)	NCT01989754	Worldwide	T2D	Canagliflozin/placebo	2907/2905	108.0	All-cause death, MACE, CV death, HHF, MI, stroke	Multicenter, double-blind RCT
Jardine et al. (19)	NCT02065791	Worldwide	T2D, CKD	Canagliflozin/placebo	2202/2199	135.6	All-cause death, MACE, CV death, HHF	Multicenter, double-blind RCT
Nassif et al. (27)	NCT02653482	US	HFrEF	Dapagliflozin/placebo	131/132	12*	All-cause death, CV death, MI, stroke	Multicenter, double-blind RCT
Wiviott et al. (7)	NCT01730534	Worldwide	T2D, ACD or were at risk for ACD	Dapagliflozin/placebo	8582/8578	219.0	All-cause death, MACE, CV death, HHF, MI, stroke	Multicenter, double-blind RCT
Bhatt et al. (20)	NCT03315143	Worldwide	T2D, CKD, risks for ACD	Sotagliflozin/placebo	5292/5292	68.6	All-cause death, MACE, CV death, HHF	Multicenter, double-blind RCT
Bhatt et al. (21)	NCT03521934	Worldwide	T2D who were recently HHF	Sotagliflozin/placebo	608/614	38.6	All-cause death, MACE, CV deaths, HHF	Multicenter, double-blind RCT
Cannon et al. (22)	NCT01986881	Worldwide	T2D and ACD	Ertugliflozin/placebo	5499/2747	156.4	All-cause death, MACE, CV death, HHF, MI, stroke	Multicenter, double-blind RCT
Heerspink et al. (32)	NCT03036150	Worldwide	CKD, with or without T2D	Dapagliflozin/placebo	2152/2152	125.1	CV death, all-cause death, HHF	Multicenter, double-blind RCT
Packer et al. (28)	NCT03057977	Worldwide	HFrEF	Empagliflozin/placebo	1863/1867	67.8	All-cause death, CV death, HHF	Multicenter, double-blind RCT
Petrie et al. (23)	NCT03036124	Worldwide	HFrEF with or without diabetes	Dapagliflozin/placebo	2373/1371	78.2	All-cause death, CV death, HHF	Multicenter, double-blind RCT
Santos-Gallego et al. (29)	NCT03485222	US	Non-diabetic HFrEF.	Empagliflozin/placebo	42/42	26.1*	All-cause death, CV death	Single-center, double-blind RCT
Anker et al. (12)	NCT03057951	Worldwide	HFpEF	Empagliflozin/placebo	2997/2991	85.7	All-cause death, CV death, HHF	Multicenter, double-blind RCT
Nassif et al. (13)	NCT03030235	US	HFpEF	Dapagliflozin/placebo	162/162	12*	All-cause death, MI, stroke	Multicenter, double-blind RCT

Main characteristics of included RCTs. RCT, randomized controlled trial; SGLT2, sodium-glucose cotransporter 2; T2D, type 2 diabetes; CKD, chronic kidney disease; HFrEF, heart failure with reduced ejection fraction; ACD, atherosclerotic cardiovascular disease; HHF, hospitalization for heart failure; HFpEF, HF with preserved ejection fraction; MACE, major adverse cardiac events; CV death, cardiovascular death; MI, myocardial infarction. *The treatment time of SGLT2 inhibitors or placebo was reported because three RCTs lacked of following up after the treatment stopped.

**FIGURE 2:**
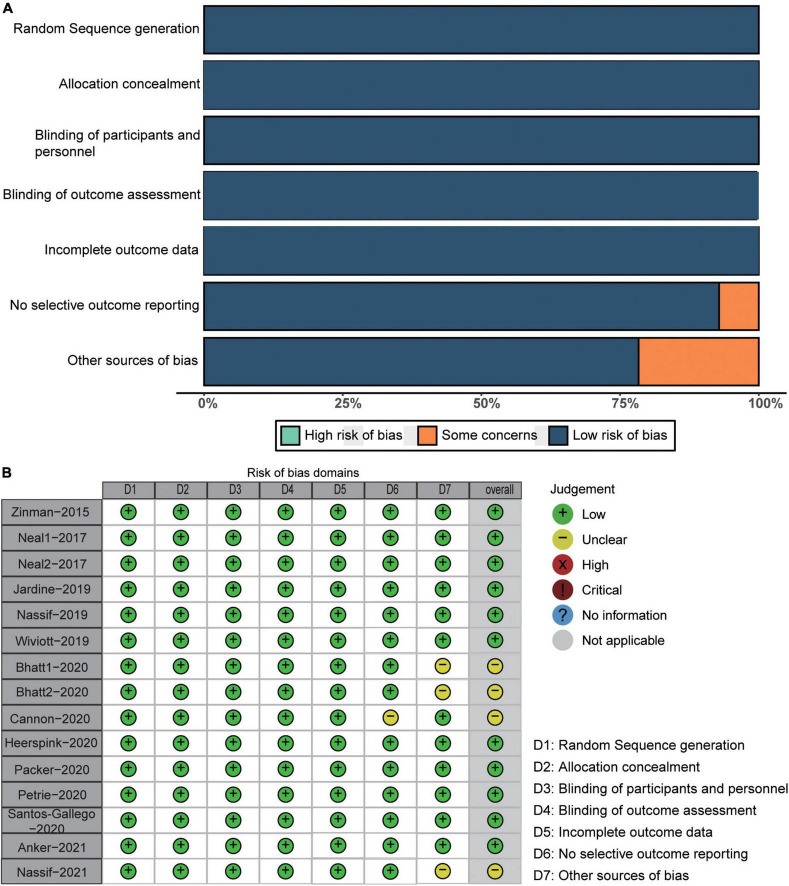
Risk of bias plot. **(A)** Risk of bias summary; **(B)** risks of bias of each included study.

We assessed the methodological quality of the included studies using the Cochrane Collaborative Assessment Tool. Figure 2 shows the methodological quality assessment results of each included RCT.

### Cardiovascular outcomes

**FIGURE 3:**
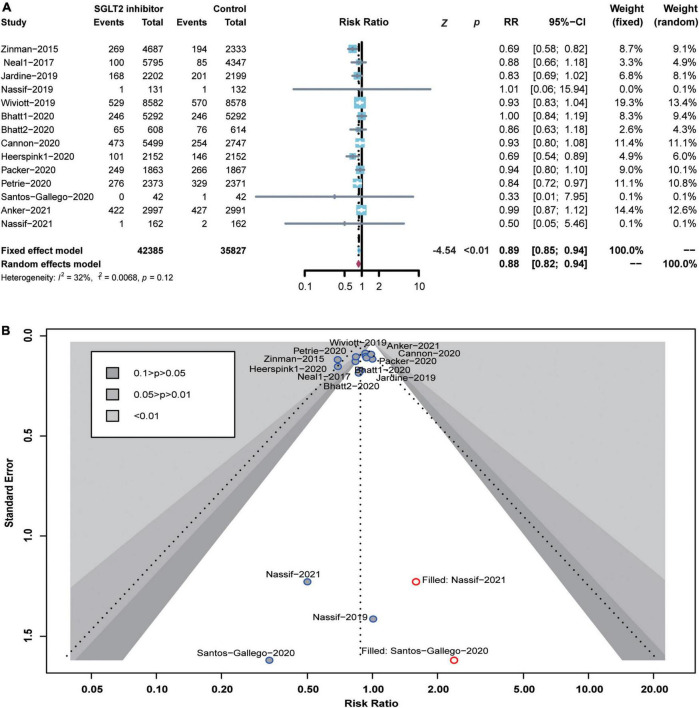
Forest plot of all-cause death and the detection of publication bias. **(A)** Forest plot of all-cause death; **(B)** funnel chart of trimming and filling.

In this study, each included RCT reported the number of all-cause deaths. Compared to the control group, the SGLT2 inhibitor significantly reduced the risk of all-cause death by 11% (RR 0.89; 95% CI: 0.85–0.94; *p* for heterogeneity 0.12; *I*^2^ = 32%; *p* < 0.01) (Figure 3A). Here, *p* > 0.05 in the Peters test indicates no publication bias. However, the funnel plots showed an asymmetric distribution of included RCTs. After trimming and filling, the funnel plots were filled with two unpublished studies in the blank area of no statistical significance. Of note, *p* < 0.05 was consistent before and after trimming and filling, indicating the robustness of our results or no risk of bias (Figure 3B).

**FIGURE 4:**
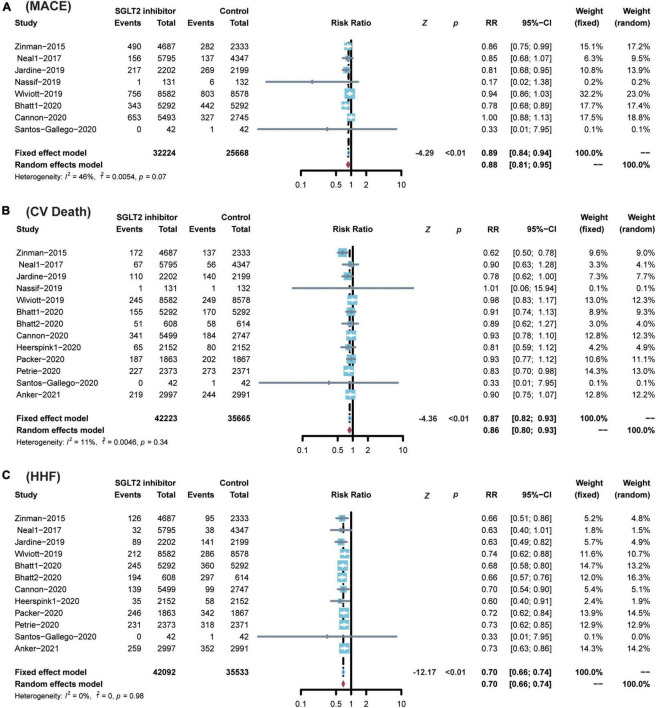
Comparison of SGLT2 inhibitor vs. control group on the risks of **(A)** MACE; **(B)** CV death; **(C)** HHF. SGLT2, Sodium-glucose cotransporter 2; MACEs, major adverse cardiac events; CV death, cardiovascular death; HHF, hospitalization for heart failure.

Compared with placebo, the administration of SGLT2 inhibitors significantly reduced the risk of CV death by 13% (RR 0.87; 95% CI: 0.82–0.93; *p* for heterogeneity 0.34; *I*^2^ = 11%; *p* < 0.01) (Figure 4), the risk of MACE by 11% (RR 0.89; 95% CI: 0.84–0.94; *p* for heterogeneity 0.07; *I*^2^ = 46%; *p* < 0.01) (Figure 4), and the risk of HF by 30% (RR 0.70; 95% CI: 0.66–0.74; *p* for heterogeneity 0.98; *I*^2^ = 0%; *p* < 0.01) (Figure 4).

However, SGLT2 inhibitors showed no advantage over placebo in reducing the risk of MI and stroke (MI: RR 0.92; 95% CI: 0.83–1.01; *p* for heterogeneity 0.44; *I*^2^ = 0%; *p* = 0.06 and stroke: RR 1.07; 95% CI: 0.94–1.22; *p* for heterogeneity 0.61; *I*^2^ = 0%; *p* = 0.29) (Supplementary Figure 1).

### Subgroup analysis

#### Diabetes

**FIGURE 5:**
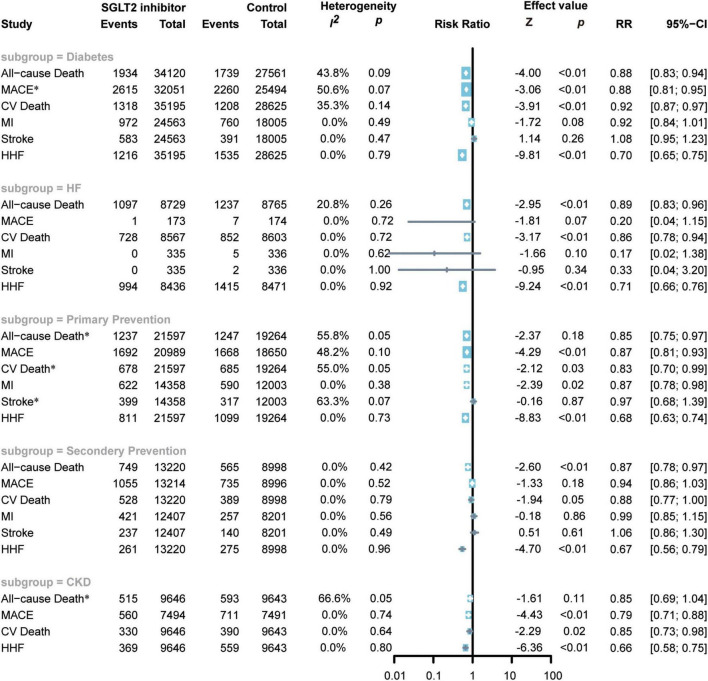
Forest plot of subgroup of subjects with diabetes, HF, ACD, high risks of ACD, and CKD. SGLT2, Sodium-glucose cotransporter 2; MACEs, major adverse cardiac events; CV death, cardiovascular death, MI, myocardial infarction; HF, heart failure; HHF, hospitalization for heart failure. *Random-effects model.

A total of nine studies including 63,820 participants reported diabetes outcomes (7, 8, 18–24). SGLT2 inhibitor administration was associated with a reduced risk of all-cause death (RR 0.88; 95% CI: 0.83–0.94; *p* for heterogeneity 0.09; *I*^2^ = 44%; *p* < 0.01) (Figure 5), MACEs (random model RR 0.88; 95% CI: 0.81–0.95; *p* for heterogeneity 0.07; *I*^2^ = 51%; *p* < 0.01) (Figure 5), CV death (RR 0.86; 95% CI: 0.79–0.93; p for heterogeneity 0.14; *I*^2^ = 35%; *p* < 0.01) (Figure 5), and HF hospitalization (RR 0.70; 95% CI: 0.65–0.75; *p* for heterogeneity 0.79; *I*^2^ = 0%; *p* < 0.01) (Figure 5) in diabetic patients compared to placebo.

However, compared to the control group, the use of SGLT2 inhibitors in diabetic patients did not show an effect in reducing the risk of MI (RR 0.92; 95% CI: 0.84–1.01; *p* for heterogeneity 0.49; *I*^2^ = 0%; *p* = 0.09) or stroke (RR 1.08; 95% CI: 0.95–1.23; *p* for heterogeneity 0.47; *I*^2^ = 0%; *p* = 0.26) (Figure 5).

#### Primary prevention of atherosclerotic cardiovascular disease

A total of six RCTs including 40,861 subjects reported the cardiovascular outcomes of the administration of SGLT2 inhibitors in patients with high risks of ACD (7, 8, 20, 21, 25, 26). Fewer patients with confirmed CAD in DECLARE-TIMI 58, EMPA-REG OUTCOME, and SOLOIST-WHF were noted compared with patients with high risks of CAD, so these three RCTs were included in the primary prevention subgroup (7, 8, 21). SGLT2 inhibitors reduced the risk of all-cause death by 15% (random: RR 0.85; 95% CI: 0.75–0.97; *p* for heterogeneity 0.05; *I*^2^ = 56%; *p* < 0.01) (Figure 5), MACEs by 13% (RR 0.87; 95% CI: 0.81–0.93; *p* for heterogeneity 0.10; *I*^2^ = 48%; *p* < 0.01) (Figure 5), CV death by 17% (random: RR 0.83; 95% CI: 0.70–0.99; *p* for heterogeneity 0.05; I^2^ = 55%; *p* < 0.01) (Figure 5), MI by 13% (RR 0.87; 95% CI: 0.78–0.98; *p* for heterogeneity 0.38; *I*^2^ = 0%; *p* = 0.02) (Figure 5), and HHF by 32% (RR 0.68; 95% CI: 0.63–0.74; *p* for heterogeneity 0.73; *I*^2^ = 0%; *p* < 0.01) (Figure 5).

However, SGLT2 inhibitors did not exhibit superiority in reducing the risk of stroke (random: RR 0.97; 95% CI: 0.68–1.39; *p* for heterogeneity 0.07; *I*^2^ = 63%; *p* = 0.66) (Figure 5).

#### Secondary prevention of atherosclerotic cardiovascular disease

Four RCTs enrolled 22218 subjects and reported the cardiovascular outcomes of applying SGLT2 inhibitors to patients with ACD (18, 22, 25, 26). The CANVAS RCTs also included patients with two or more cardiovascular risks or cardiovascular atherosclerotic diseases. The number of patients with the latter was greater than that of the former, so we included this study in the secondary prevention of CHD (18). SGLT2 inhibitors reduced the risk of all-cause death by 13% (RR 0.87; 95% CI: 0.78–0.97; p for heterogeneity 0.42; *I*^2^ = 0%; *p* = 0.01) (Figure 5) and the risk of CV death by 12% (random: RR 0.88; 95% CI: 0.77–1.00; *p* for heterogeneity 0.79; *I*^2^ = 0%; *p* = 0.05) (Figure 5), and the risk of HHF (RR 0.67; 95% CI: 0.56–0.79; *p* for heterogeneity 0.96; *I*^2^ = 0%; *p* < 0.01) (Figure 5).

The administration of SGLT2 inhibitors to patients with ACD did not exhibit a significant effect in reducing the risk of MACEs (RR 0.94; 95% CI: 0.86–1.03; *p* for heterogeneity 0.52; *I*^2^ = 0%; *p* = 0.18) (Figure 5), MI (RR 0.99; 95% CI: 0.85–1.15; *p* for heterogeneity 0.56; *I*^2^ = 0%; *p* = 0.86) (Figure 5), or stroke (RR 1.06; 95% CI: 0.86–1.30; *p* for heterogeneity 0.49; *I*^2^ = 0%; *p* = 0.61) (Figure 5).

#### Heart failure

Nine RCTs enrolled 17494 patients with HF (12, 13, 21, 23, 27–31). SGLT2 inhibitors significantly reduced the incidence of all-cause death (RR 0.89; 95% CI: 0.83–0.96; *p* for heterogeneity 0.26; *I*^2^ = 21%; *p* < 0.01) (Figure 5), CV death (RR 0.86; 95% CI: 0.78–0.94; *p* for heterogeneity 0.72; *I*^2^ = 0%; *p* < 0.01) (Figure 5), and HHF (RR 0.71; 95% CI: 0.66–0.76; *p* for heterogeneity 0.92; *I*^2^ = 0%; *p* < 0.01) (Figure 5) but not reduce the incidence of MACEs (RR 0.20; 95% CI: 0.04–1.15; *p* for heterogeneity 0.72; *I*^2^ = 0%; *p* = 0.07) (Figure 5), MI (RR 0.17; 95% CI: 0.02–1.38; *p* for heterogeneity 0.62; *I*^2^ = 0%; *p* = 0.10) (Figure 5), or stroke (RR 0.33; 95% CI: 0.04–3.20; *p* for heterogeneity 1.00; *I*^2^ = 0%; *p* = 0.34) (Figure 5).

#### Chronic kidney disease

Three RCTs included 19289 patients with CKD (19, 20, 32). The administration of SGLT2 inhibitors can significantly reduce the risk of MACEs (RR 0.79; 95% CI: 0.71–0.88; *p* for heterogeneity 0.74; *I*^2^ = 0%; *p* < 0.01) (Figure 5), CV death (RR 0.85; 95% CI: 0.73–0.98; *p* for heterogeneity 0.64; *I*^2^ = 0%; *p* = 0.02) (Figure 5), HF (RR 0.66; 95% CI: 0.58–0.75; *p* for heterogeneity 0.80; *I*^2^ = 0%; *p* < 0.01) (Figure 5), but not reduce the risk of all-cause death (random: RR 0.84; 95% CI: 0.69–1.04; *p* for heterogeneity 0.05; *I*^2^ = 67%; *p* = 0.11) (Figure 5).

### Safety events

**FIGURE 6:**
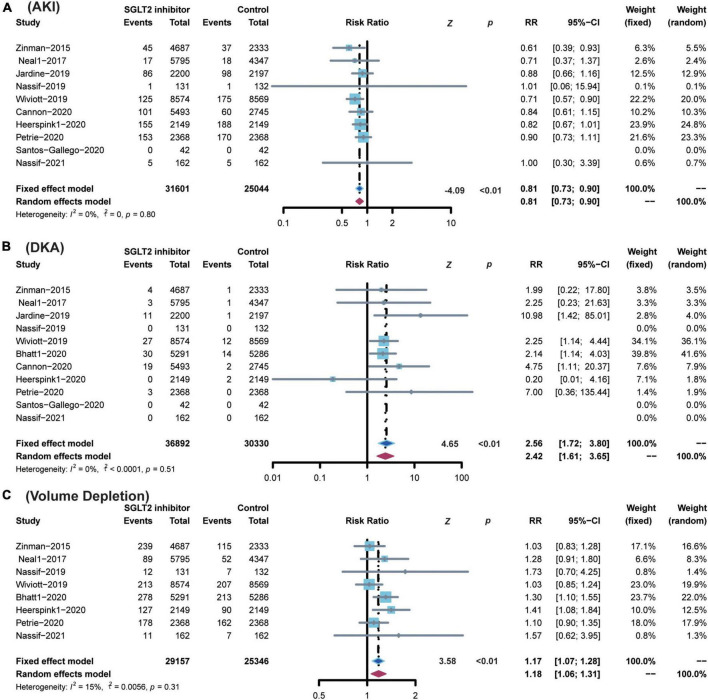
Comparison of SGLT2 inhibitor vs. control group on the risks of **(A)** AKI; **(B)** DKA; **(C)** Volume Depletion. SGLT2, sodium-glucose cotransporter 2; AKI, acute kidney injury; DKA, diabetic ketoacidosis.

SGLT2 inhibitors showed superiority in reducing the risk of AKI (RR 0.81; 95% CI: 0.73–0.90; *p* for heterogeneity 0.80; *I*^2^ = 0%; *p* < 0.01) (Figure 6). SGLT2 inhibitors enhanced the risk of DKA (RR 2.56; 95% CI: 1.72–3.80; *p* for heterogeneity 0.51; *I*^2^ = 0%; *p* < 0.01) (Figure 6) and volume depletion (RR 1.17; 95% CI: 1.07–1.28; *p* for heterogeneity 0.31; *I*^2^ = 15%; *p* < 0.01) (Figure 6).

SGLT2 inhibitors did not show any significant effect on the incidence of amputation (RR 1.12; 95% CI: 0.97–1.30; *p* for heterogeneity 0.63; *I*^2^ = 0%; *p* = 0.12), bone fracture (RR 1.04; 95% CI: 0.96–1.13; *p* for heterogeneity 0.83; *I*^2^ = 0%; *p* = 0.36), hypoglycemia (RR 0.96; 95% CI: 0.92–1.01; *p* for heterogeneity 0.18; I2 = 31%; *p* = 0.17), thromboembolic events (RR 0.83; 95% CI: 0.59–1.17; *p* for heterogeneity 0.82; *I*^2^ = 0%; *p* = 0.28), or urinary tract infection (RR 1.05; 95% CI: 0.99–1.12; *p* for heterogeneity 0.24; *I*^2^ = 27%; *p* = 0.09) (Supplementary Figure 2).

## Discussion

Fourteen RCTs were included in this meta-analysis to assess the effect of SGLT2 inhibitors on cardiovascular outcomes and safety events. Our research integrated the cardiovascular outcomes of SGLT2 inhibitors in all subjects and compared the cardiovascular outcomes of different subgroups of patients receiving SGLT2 inhibitors according to the subjects’ past medical history. We found that SGLT2 inhibitor reduced all-cause death, MACE, CV death, and HHF but did not have a significant effect on MI and stroke. SGLT2 inhibitors also reduced AKI but significantly increased the risk of DKA and volume depletion.

Our subgroup analysis found that SGLT2 inhibitors reduce the risk of MACEs, CV death, all-cause death, and HHF in diabetic patients; reduce the risk of MACEs, CV death, MI, all-cause death, and HHF in people with a high risk of ACD; reduce the risk of all-cause death, CV death, HHF and other risks of patients with ACD and HF; and reduce the risk of MACEs, CV death, HHF and other risks in CKD patients.

The cardiovascular benefits of SGLT2 inhibitors have been further explored since the FDA approved them as an effective therapy for the treatment of diabetes. The results of our meta-analysis are highly consistent with previous meta-analyses, both confirming that SGLT2 inhibitors reduce cardiovascular risk and exhibit superiority in diabetic patients (13). This finding is potentially noted because diabetes is an independent risk factor for cardiovascular disease, and SGLT2 inhibitors reduce blood glucose and further reduce cardiovascular risk. However, some studies have noted that hyperglycemia mainly causes microvascular complications. However, macrovascular complications, such as MI and stroke caused by hyperglycemia, may only occur after decades (33). Therefore, the cardiovascular benefit of SGLT2 inhibitors may occur independently of the hypoglycemic effect.

SGLT2 inhibitors play a major role in promoting natriuresis and glucosuria, resulting in osmotic diuresis and concentrated blood volume for cardiovascular benefits. Blood concentrations (presumably secondary to volume contraction) accounted for approximately 50% of the observed cardiovascular benefits (8). In addition, SGLT2 can increase ketone levels in the blood circulation while promoting the rate of ketone oxidation and energy metabolism in the heart. Santos-Gallego et al. (34) found that empagliflozin can reduce the risk of cardiac adverse remodeling and HF in a porcine model of HF by improving cardiac energetics (35). Studies have also shown that SGLT2 inhibitors can improve mitochondrial respiratory function in diabetic rats, which may also help improve energy production in the heart (36). The anti-inflammatory properties of SGLT2 inhibitors were associated with reduced inflammatory molecular processes, such as extracellular matrix turnover and fibrosis (37). Canagliflozin and dapagliflozin reduced or improved inflammation in diabetic patients (38). SGLT2 inhibitors also significantly improved the left ventricular mass index changes and inhibited ventricular remodeling in patients with diabetes and ACD after six months of treatment (39). Emperor-Reduced enrolled patients with HFrEF and demonstrated that empagliflozin reduced the rate of HHF, and this clinical benefit was consistent in diabetic and non-diabetic patients. Emperor-Preserved enrolled patients with HF of preserved ejection fraction (HFpEF) and found that empagliflozin performed consistently in patients with HFpEF and those with HFrEF. However, none of the trials found that empagliflozin reduced the risk of cardiovascular death. Kumar et al. further confirmed that SGLT2 inhibitors can reduce cardiovascular risk in patients with heart failure, which is consistent with our findings (40).

Multiple previous studies have focused on the cardiovascular benefits of SGLT2 inhibitors in diabetic patients. However, evidence on the cardiovascular benefits of SGLT2 inhibitors in patients at high risk of ACD and patients with confirmed ACD is lacking. DECLARE-TIMI58 is the largest clinical RCT to date investigating the clinical benefits of SGLT2 inhibitors for the primary and secondary prevention of ACD. This study noted that SGLT2 can effectively reduce the risk of CV death and HHF in patients with diabetes and ACD or high-risk ACD.

VETRIS CV included all patients with diabetes and ACD and found that SGLT2 only reduced the hospitalization rate of HF and had no obvious benefit for cardiovascular events, such as MACEs, CV death, and MI (22). A meta-analysis by Zelniker et al. noted that SGLT2 inhibitor administration in the secondary prevention of ACD reduced MACEs and CV death combined with HHF, but this benefit was not observed for primary prevention (9). This study found that SGLT2 inhibitors reduce the risk of MACEs in primary and secondary prevention, which is consistent with Zelniker’s meta-analysis. However, our study found that SGLT2 not only reduces the risk of hospitalization and cardiovascular death in primary and secondary prevention but also reduces cardiovascular all-cause mortality. This difference may be related to the size of the included population. Compared with the three trials included in Zelniker’s meta-analysis, 15 studies were included in our research to reduce the risk of drawing incorrect conclusions (9). However, type 2 diabetes was diagnosed in every study population, both in the subgroups of patients with high risk of ACD and confirmed ACD. Therefore, further studies should be performed to include non-diabetic patients to explore the effect of SGLT2 inhibitors on patients with a high risk of ACD and verify ACD in non-diabetic states.

Given that SGLT2 inhibitors exert osmotic diuretic effects by reducing glucose and sodium reabsorption through SGLT2 inhibition, the link between SGLT2 inhibitors and renal function has been further explored. Some studies have found that SGLT2 inhibitors can dilate efferent arterioles and contract afferent arterioles to repair bulbar feedback to protect renal function. Compared to placebo, SGLT2 inhibitors effectively promote the recovery of the glomerular filtration rate to baseline levels (41–43). SGLT2 increases the glomerular filtration rate. In addition, the recovered glomerular filtration rate also continues to remain stable after the drug is discontinued (44).

CREDENCE is the world’s first Renal Outcome Trial (ROT) of a hypoglycemic agent, aiming to investigate the effect of canagliflozin on renal endpoints in patients with type 2 diabetes (T2DM) and chronic kidney disease (CKD) (19). The results confirmed that in patients with T2DM and CKD, canagliflozin significantly reduced the risk of renal events and cardiovascular events with a good safety profile. These results are consistent with previous systematic reviews and meta-analyses that included patients with T2DM and CKD treated with various SGLT2 inhibitors (45). Based on the results of the CREDENCE trial, the FDA approved canagliflozin to treat diabetic nephropathy and confirmed that canagliflozin exhibit a good ability to reduce the risk of HHF in patients with type 2 diabetes and diabetic nephropathy. The Dapagliflozin and Prevention of Adverse Outcomes in Chronic Kidney Disease (DAPA-CKD) trial showed that the SGLT2 inhibitor Dapagliflozin improved renal and cardiovascular outcomes in patients with CKD regardless of the presence of T2DM (12). Previous meta-analyses have shown that SGLT2 inhibitors reduce the risk of renal dialysis or death in patients with T2DM and also provide protection against AKI, which was also demonstrated in our study (46).

In addition to reducing the risk of AKI, SGLT2 inhibitors also increase the risk of DKA and volume depletion. The elevated urine glucose in diabetic patients leads to increased bacterial adhesion to the urinary tract. And because of the weakened cellular and humoral immune response in diabetic patients, the chance of urinary tract infections increases 1.5–4 times compared to ordinary people (47). In 2015, the FDA warned about the increased risk of severe urinary tract infections with SGLT2 inhibitors (48). However, recent clinical RCTs have not provided conclusive evidence that SGLT2 inhibitors increase the risk of urinary tract infection (7, 8, 18). Our findings are consistent with a previous meta-analysis that included 86 RCTs, indicating that SGLT2 inhibitors were not significantly associated with urinary tract infections (49). In addition, SGLT2 inhibitors have previously reported side effects such as fracture, and amputation, but this study did not confirm this, which is consistent with a previous meta-analysis (45). SGLT2 inhibitors are associated with the occurrence of DKA, and a blood glucose level < 14 mmol/L, which we refer to as “DKA with lower-than-anticipated glucose levels.” Individuals with DKA, including those with euglycemia, may exhibit symptoms of thirst, polyuria, nausea, vomiting, abdominal pain, confusion, sense of air hunger (Kussmaul’s breathing), fever, and fruity odor on the breath (acetone), as well as indications of any of the precipitating factor(s). Therefore, we need monitor and prevent the occurrence of DKA in our clinical applications and actively employ the positive benefits of an SGLT2 inhibitor in reducing blood glucose and protecting the cardiovascular system. We believe that SGLT2 inhibitors should be discontinued as soon as symptoms of DKA are found in patients regardless of whether these symptoms are induced by SGLT2 inhibitors. In addition, to reduce the risk of DKA in patients with T2DM, SGLT2 inhibitors should be discontinued in patients with acute trauma or stress after major surgery.

## Limitations

The heterogeneity among the RCTs included in this study was small. The funnel plot after the cut-and-fill method of the Cochrane quality assessment tool suggested no risk of bias. At the same time, we used a random-effects model and a fixed-effects model to calculate the statistical results to verify the robustness of the outcomes. However, this study also has certain limitations. First, the number of participants in three RCTs was small and these three studies reported outcomes immediately upon cessation of drug treatment; thus, long-term follow-up was not available (13, 27, 29). Second, fewer RCTs report the corresponding outcome in specific subgroups; thus, further large-sample RCTs are required. Third, most patients with diagnosed diabetes received primary hypoglycemic therapy, which may affect the cardiovascular benefits of SGLT2 inhibitors.

## Conclusion

SGLT2 inhibitors exhibit superiority in reducing the risk of cardiovascular outcomes such as CV death, MACEs, and HHF in adults and reducing the incidence of all-cause death, AKI, and DKA. In addition, SGLT2 inhibitors may also reduce the risk of MI as primary prevention of CHD, as well as the risk of MACEs, CV death, all-cause death, and HHF as secondary prevention.

## Data availability statement

The original contributions presented in this study are included in the article/Supplementary material, further inquiries can be directed to the corresponding authors.

## Author contributions

CG and S-CS contributed to the study concept and design of the study. J-JS and J-YZ collected the data. S-CS, KZ, and LZ conducted the analysis and presentation of data for the work. CG and S-CS drafted the manuscript. HG and L-KM were responsible for guiding the study, editing, and reviewing the manuscript. All authors have read and approved the final manuscript.

## Footnotes

1
https://www.crd.york.ac.uk/prospero/

## Supplementary material

Supplementary Figure 1 Comparison of SGLT2 inhibitor vs. control group on the risks of **(A)** MI; **(B)** stroke. SGLT2, sodium-glucose cotransporter 2; MI, myocardial infarction.

Supplementary Figure 2 Comparison of SGLT2 inhibitor vs. control group on the risks of **(A)** amputation; **(B)** bone fraction; **(C)** hypoglycemia; **(D)** thromboembolic events; **(E)** urinary tract infection. SGLT2, sodium-glucose cotransporter 2.
