# Supplementary figures and images for: Association of sodium-glucose cotransporter 2 inhibitors with cardiovascular outcome and safety events: A meta-analysis of randomized controlled clinical trials
Source: Front Cardiovasc Med. 2022 Oct 14;9:926979. doi: 10.3389/fcvm.2022.926979 (PMC9613919; doi:10.3389/fcvm.2022.926979)

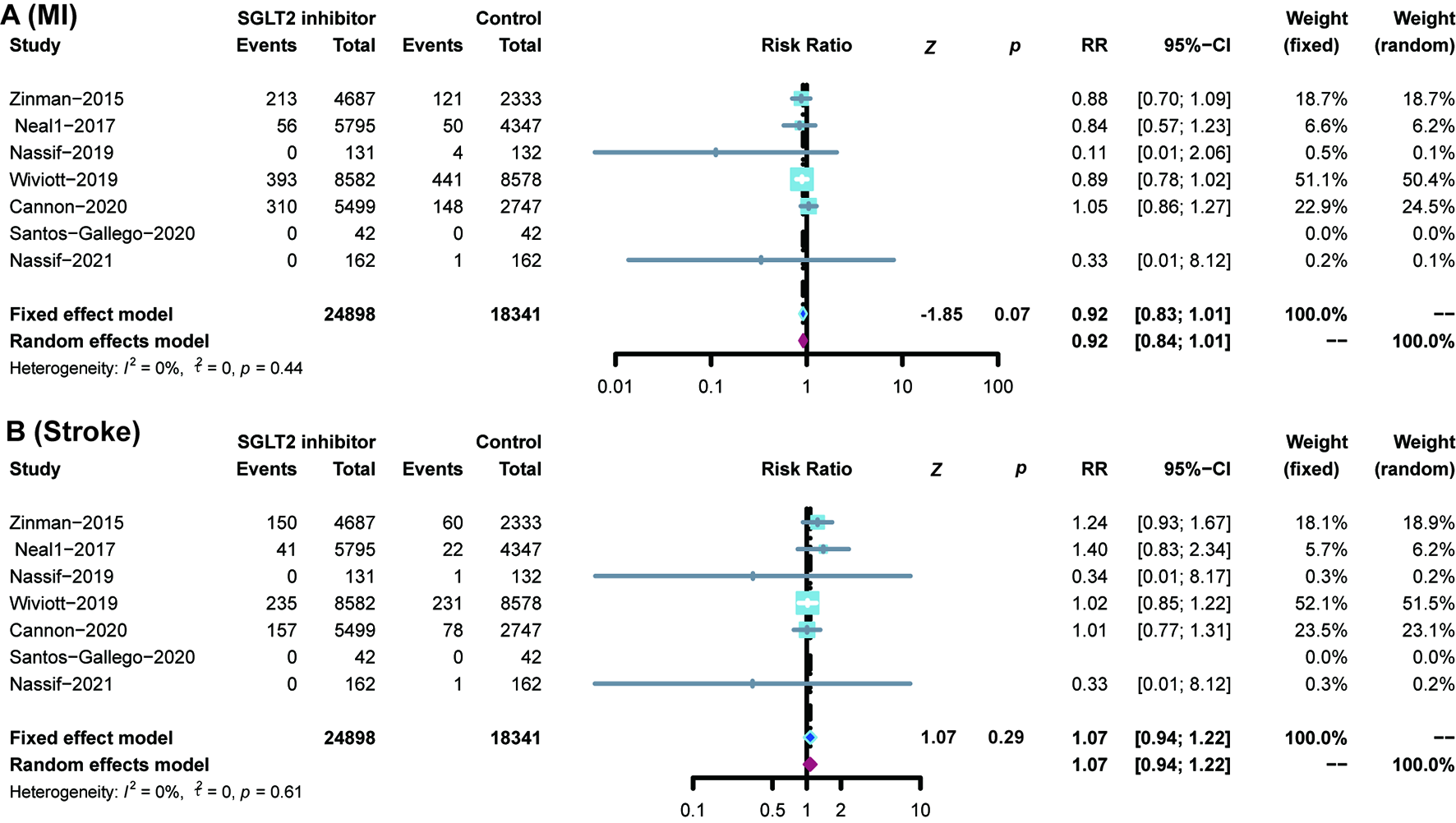

Supplement: Supplementary Figure 1 — Comparison of SGLT2 inhibitor vs. control group on the risks of (A) MI; (B) stroke. SGLT2, sodium-glucose cotransporter 2; MI, myocardial infarction. [file Image_1.TIF]

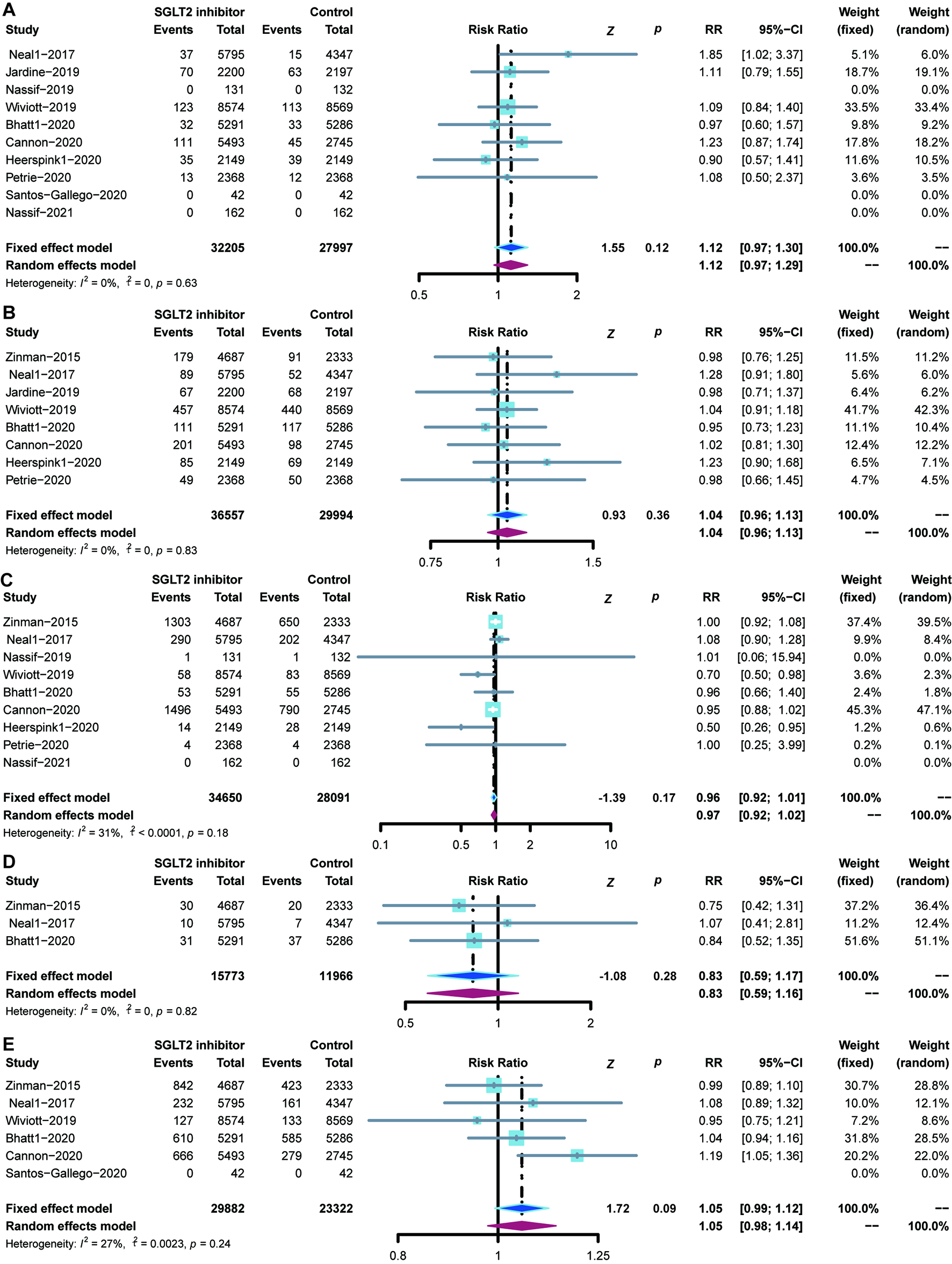

Supplement: Supplementary Figure 2 — Comparison of SGLT2 inhibitor vs. control group on the risks of (A) amputation; (B) bone fraction; (C) hypoglycemia; (D) thromboembolic events; (E) urinary tract infection. SGLT2, sodium-glucose cotransporter 2. [file Image_2.TIF]
